# Cinquième cycle mensuel de la chimioprévention du paludisme saisonnier chez les enfants de 3 à 59 mois dans la région de Zinder, Niger: approche de son impact

**DOI:** 10.11604/pamj.2026.53.171.48880

**Published:** 2026-04-22

**Authors:** Almoustapha Mahamane Wazodan, Mahaman Moustapha Lamine, Ibrahim Alkassoum, Mahamadou Doutchi, Éric Adehossi, Léon Savadogo

**Affiliations:** 1 Ecole Doctorale de Sciences et Techniques, Université Nazi Boni, Bobo-Dioulasso, Burkina Faso,; 2 Direction de Service de Santé et d´Action Sociale de la Garde Nationale du Niger, Niamey, Niger,; 3 Lab-EGB2S, CFRMT-UAM, Département des Sciences Biologiques, Université André Salifou, Zinder, Niger,; 4 Centre de Formation et de Recherche en Médecine Tropicale, Faculté des Sciences de la Santé, Niamey, Niger,; 5 Département de Médecine et Spécialités Médicales, Faculté des Sciences de la Santé, CFRMT-UAM, Université André Salifou, Zinder, Niger,; 6 Institut Supérieur des Sciences de la Santé, Université Nazi Boni, Bobo-Dioulasso, Burkina Faso

**Keywords:** Impact, cinquième passage, chimioprévention du paludisme saisonnier, Niger, Impact, fifth cycle, seasonal malaria chemoprevention, Niger

## Abstract

L'ajout d'un cinquième cycle mensuel à la chimioprévention saisonnière du paludisme (CSP) a été proposé afin de prolonger la protection pendant les saisons de transmission prolongées au Sahel. Cette étude a évalué l'effet de l'introduction d'un cinquième cycle de CSP sur l'incidence du paludisme et sur l'utilisation des services de diagnostic et de traitement du paludisme dans le district sanitaire de Takieta, au Niger. Nous avons mené une étude quasi-expérimentale, non randomisée, avant-après, avec un groupe de comparaison (conception « ici-ailleurs ») en utilisant les données de santé courantes de 19 centres de santé intégrés répartis dans deux districts sanitaires entre 2020 et 2023. Neuf centres de santé ont mis en œuvre cinq cycles mensuels de CSP (CSP5), tandis que dix centres de santé ont poursuivi la stratégie standard à quatre cycles. Les données ont été extraites du système national d'information sanitaire. Un modèle à effets fixes de différence dans les différences (DiD) a été appliqué pour estimer l'impact du cinquième cycle de SMC sur l'incidence du paludisme, l'utilisation des tests de diagnostic rapide (TDR), la positivité des TDR et la consommation de combinaisons thérapeutiques à base d'artémisinine (ACT). L'introduction d'un cinquième cycle de SMC a été associée à une réduction significative de l'incidence du paludisme, correspondant à une diminution de 2,44 cas pour 1 000 habitants pendant la période d'intervention. Cette réduction s'est accompagnée d'une amélioration de 2,99% des performances des services de soins et d'une baisse significative des taux de positivité des TDR (p = 0,003). Cependant, les coefficients de différence dans les différences pour la consommation de TDR (158,59) et la consommation d'ACT (153,05) n'étaient pas statistiquement significatifs, ce qui indique que le SMC5 n'a pas eu d'impact mesurable sur la réduction de l'utilisation des tests diagnostiques ou des traitements antipaludiques. L'ajout d'un cinquième cycle mensuel de chimioprévention saisonnière du paludisme a été associé à une réduction de l'incidence du paludisme dans les districts étudiés pendant la période d'intervention. Cependant, aucune preuve d'une réduction correspondante de la consommation de TDR ou d'ACT n'a été trouvée. Ces résultats suggèrent que l'extension de la couverture de la SMC d'un mois supplémentaire peut réduire la charge du paludisme sans modifier de manière significative l'utilisation des services de diagnostic ou de traitement dans les établissements de santé.

## Abstract (en)

The addition of a fifth monthly cycle to seasonal malaria chemoprevention (SMC) has been proposed to extend protection during prolonged transmission seasons in the Sahel. This study evaluated the effect of introducing a fifth SMC cycle on malaria incidence and on the utilization of malaria diagnostic and treatment services in the Takieta health district, Niger. We conducted a quasi-experimental, non-randomized, before-and-after study with a comparison group (“here-elsewhere” design), using routine health data from 19 integrated health centers across two health districts between 2020 and 2023. Nine health centers implemented five monthly SMC cycles (SMC5), while ten continued the standard four-cycle strategy. Data were extracted from the national health information system. A fixed-effects difference-in-differences (DiD) model was applied to estimate the impact of the fifth SMC cycle on malaria incidence, rapid diagnostic test (RDT) utilization, RDT positivity rates, and artemisinin-based combination therapy (ACT) consumption. The introduction of a fifth SMC cycle was associated with a significant reduction in malaria incidence, corresponding to a decrease of 2.44 cases per 1,000 population during the intervention period. This reduction was accompanied by a 2.99% improvement in health service performance and a significant decline in RDT positivity rates (p = 0.003). However, the difference-in-differences coefficients for RDT use (158.59) and ACT use (153.05) were not statistically significant, indicating that SMC5 had no measurable impact on reducing the use of diagnostic tests or antimalarial treatments. The addition of a fifth monthly cycle of seasonal malaria chemoprevention was associated with a reduction in malaria incidence in the study districts during the intervention period. However, no corresponding reduction in RDT or ACT consumption was observed. These findings suggest that extending SMC coverage by one additional month may reduce malaria burden without significantly altering the use of diagnostic or treatment services in health facilities.

## Introduction

Le paludisme constitue un problème majeur de santé publique en termes de morbidité et de mortalité [1]. Il s'agit d´une maladie fébrile et hémolytique causée par des parasites du genre *Plasmodium*, transmise à l'homme par la piqûre de moustiques femelles infectés du genre *Anopheles* [2]. Selon l'OMS, le nombre de cas de paludisme dans le monde s´élève à 263 millions, avec 597 000 décès enregistrés en 2023, la région africaine représentant près de 95% de ces décès, majoritairement dans des contextes où l'accès aux services de prévention, de diagnostic et de traitement reste limité [3]. Les enfants de moins de cinq ans sont particulièrement touchés par cette charge sanitaire. Au Niger, le paludisme représente la première cause de consultation, d´hospitalisation et de mortalité dans les structures de santé publique [4]. Comme dans de nombreux pays sahéliens, la transmission du paludisme est fortement saisonnière, concentrée sur quelques mois de l´année. Pour faire face à cette réalité épidémiologique, l'Organisation mondiale de la Santé (OMS) a recommandé en 2012 la mise en œuvre de la chimioprévention du paludisme saisonnier (CPS) chez les enfants de moins de 5 ans vivant dans ces pays [5]. Depuis son introduction en 2013, la CPS suit un protocole d´administration mensuelle de 3 jours de sulfadoxine-pyriméthamine (SP) et d'amodiaquine (AQ) aux enfants de 3 à 59 mois durant la période de haute transmission [6]. L'expérience de sa mise en œuvre par les équipes de Médecins Sans Frontières (MSF) a été décrite dans un rapport de capitalisation [7]. D'autres études d'évaluation ont également montré que 94% des parents sont satisfaits de la chimioprévention du paludisme saisonnier. Le taux de couverture de la CPS (4 passages) est de 81,3%, avec une observance du traitement atteignant 99,2%. Aucun événement indésirable majeur n´a été enregistré [8]. Son efficacité est bien documentée par de nombreuses études en Afrique [3], avec au Niger des impacts significatifs: une réduction de 73% de l'incidence du paludisme simple et une diminution de 26,5% des formes graves [9]. Au vu de ces résultats, la CPS a été mise en œuvre dans 28 districts en 2017, puis étendue l´année suivante à la quasi-totalité du Niger, soit 60 districts sur 72. Cette extension a fait l'objet d'une évaluation révélant un taux de couverture national de 85,75% [10].

Malgré ces efforts considérables, d´énormes défis persistent pour une mise en œuvre efficace et efficiente. Parmi eux figure la difficulté d´accès à certaines zones, due à la mauvaise qualité des infrastructures et à l´instabilité politique [11]. On observe également un rebond du nombre de cas de paludisme à la fin du 4^e^ passage de la CPS, notamment dans les zones où la durée de haute transmission est longue, ce que l'intervention ne permet pas de couvrir intégralement [12]. Pour y remédier, un cinquième passage mensuel a été introduit en 2022 dans certains districts selon leur profil épidémiologique. Des expériences similaires au Mali et au Burkina Faso ont montré des résultats satisfaisants en termes de réduction de l´incidence, d´acceptation par la population et de faisabilité [13,14]. Au Niger, cette extension a également été évaluée par une étude qualitative sur la perception de la population et l´observance des doses [15]. Compte tenu des contraintes liées aux données de routine et de l'absence d´assignation aléatoire, la méthode de double différence a été retenue pour estimer l'effet du cinquième cycle. Cette approche permet de comparer les variations observées avant et après l'intervention entre les districts ayant reçu le cinquième passage et ceux n'ayant pas bénéficié de cette extension, tout en contrôlant les différences initiales et les effets temporels constants [16].

L'objectif de cette étude était d'évaluer l'impact de l'introduction d'un cinquième cycle mensuel de chimioprévention du paludisme saisonnier sur l´incidence du paludisme, l´utilisation des tests de diagnostic rapide et des combinaisons thérapeutiques à base d´artémisinine chez les enfants de 3 à 59 mois dans le district sanitaire de Takieta, au Niger.

## Méthodes

**Figure 1:**
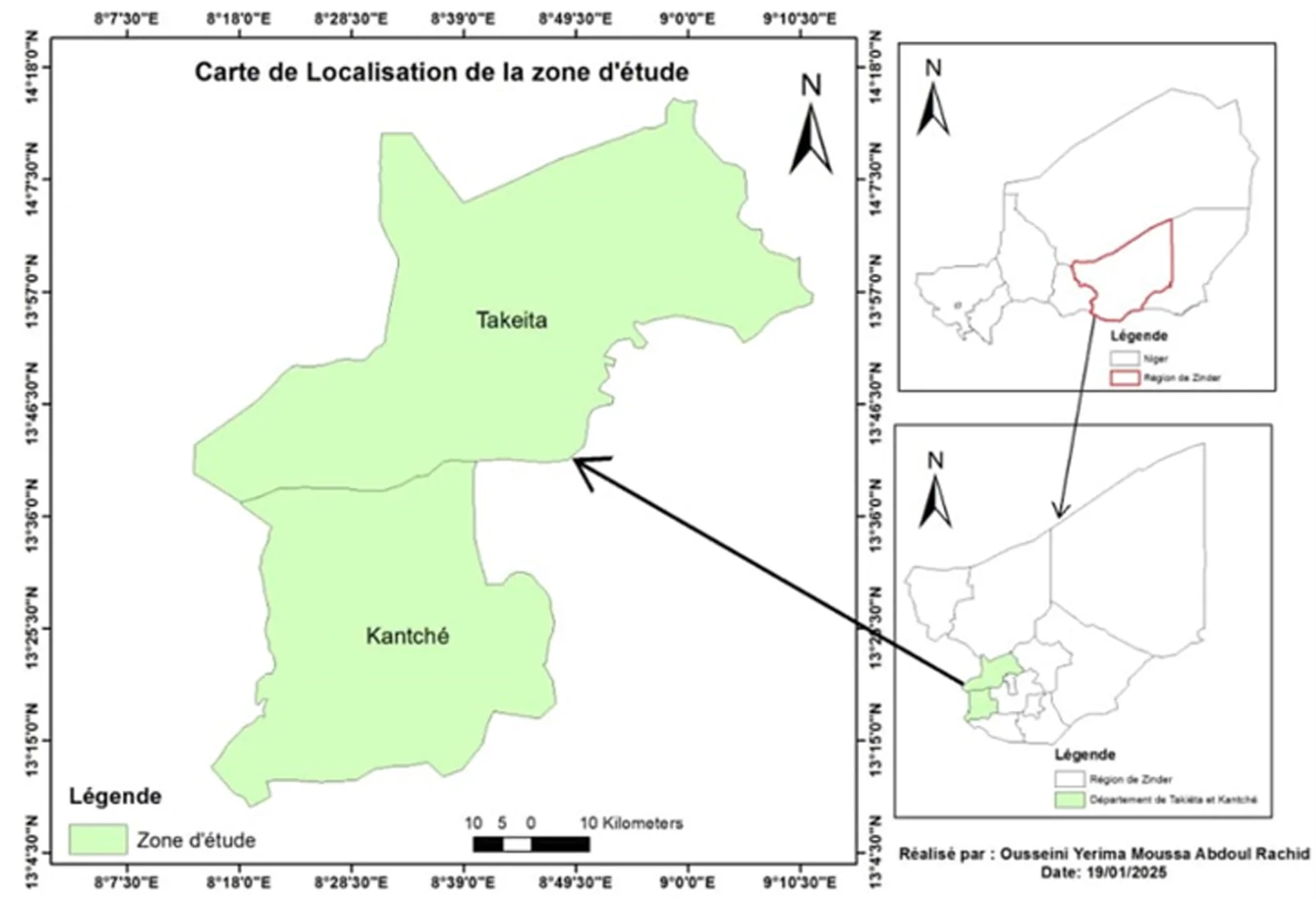
zone d'étude

**Zone d'étude:** notre étude a été effectuée sur toute l'étendue territoriale du département de Takieta et de Matameye dans la région de Zinder (Figure 1). Avec une population de 939 671 habitants dont 192 322 enfants de moins de 5 ans [17]. Le climat est de type sahélo-soudanien, avec des sols de nature sablonneuse, argilo-sableuse et limono-sableuse. La pluviométrie, caractérisée par son irrégularité et sa mauvaise répartition spatio-temporelle, présente des moyennes annuelles respectives de 665 mm et 676,2 mm [18]. Il y a deux saisons: une saison pluvieuse de mai à octobre et une saison sèche, elle-même divisée en deux périodes: l'une froide de novembre à février et l´autre chaude de mars à avril. En termes de charge palustre, le nombre de cas de paludisme traités à l’ACT pour les deux districts sanitaires est estimé à environ 173 404 et le nombre de cas de paludisme chez les enfants de moins de 5 ans est de 120 995, soit une incidence de paludisme de 669 pour 1000 [19].

**Type d'étude:** il s'agit d'une étude quasi-expérimentale de type ici-ailleurs et avant-après non randomisée sur 19 centres de santé intégrés (CSI) dont 10 sans CPS5 (groupe contrôle) et 9 avec CPS5 (groupe traité) répartis sur 2 districts sanitaires appartenant à la région de Zinder.

**Période d'étude:** du 1^er^ janvier 2020 au 31 décembre 2023, soit une durée de quatre ans (2020-2023), dont deux correspondent à la période avant la mise en œuvre de la stratégie et deux autres années de suivi post-introduction.

### Population d´étude

La population cible comprenait tous les enfants âgés de 3 à 59 mois résidant dans les zones couvertes par la CPS. Les centres de santé inclus devaient être éligibles à la CPS, disposer de données complètes et maintenir la disponibilité des intrants (médicaments et tests rapides) sans rupture de plus de sept jours pendant la période d'étude. Les centres avec des données mensuelles manquantes sur le paludisme chez les enfants de moins de cinq ans ont été exclus. Au total, 19 centres de santé intégrés (CSI) ont été inclus, 9 ont reçu le cinquième cycle de CPS (groupe traité) et 10 ont maintenu quatre cycles standards (groupe témoin). Aucune estimation de taille d´échantillon n´a été réalisée, les 19 CSI représentant la population étudiée.

### Collecte de données

Les données ont été extraites du système d'information sanitaire DHIS2 des centres de santé, comprenant le nombre de cas de paludisme confirmés par test de diagnostic rapide (TDR), le nombre de TDR réalisés, le nombre de TDR positifs et le nombre d´ACT utilisés. Les données ont été collectées mensuellement pour chaque site entre janvier 2020 et décembre 2023, incluant deux années avant et deux années après l´introduction du cinquième cycle. Le cinquième cycle de CPS (CPS5) a été administré en novembre 2022, selon le protocole standard de trois jours de sulfadoxine-pyriméthamine plus amodiaquine, aux enfants de 3 à 59 mois des districts sélectionnés afin de prolonger la protection pendant la période résiduelle de transmission.

### Définitions

L'incidence du paludisme correspond au nombre de cas confirmés par TDR rapporté à la population cible pour 1 000 habitants. L'utilisation des ACT correspond au nombre de combinaisons thérapeutiques à base d´artémisinine administrées aux enfants de 3 à 59 mois. L´utilisation des TDR correspond au nombre de tests rapides réalisés, et le TDR positif au nombre de tests confirmant une infection palustre. La CPS5 désigne l´ajout d´un cinquième passage mensuel à la CPS standard de quatre cycles.

### Analyse des données

Les données manquantes ont fait l'objet d´une imputation multiple pour celles dont le taux ne dépassait pas le seuil de 5%. Les données ont été présentées sous forme de tableaux et de graphiques. Les cas ont été cumulés par mois, année et site d'étude. L'incidence a été calculée en rapportant le nombre de cas confirmés à la population cible. Nous avons utilisé le test du Khi^2^ de Pearson et le test exact de Fisher pour déterminer l'association entre les variables catégorielles, à l'aide du logiciel Stata, version 18. Les données ont été analysées à l'aide du logiciel Stata version 18. Les données manquantes inférieures à 5% ont été imputées par imputation multiple. Les cas ont été agrégés par mois, année et site d´étude, et l'incidence a été calculée par rapport à la population cible. Les associations entre variables catégorielles ont été évaluées par le test du Khi^2^ de Pearson ou le test exact de Fisher. L'impact du cinquième cycle a été estimé via la méthode de double différence (difference-in-differences), combinant la comparaison avant-après dans le groupe CPS5 et la différence entre les groupes traités et témoins, afin d'isoler l'effet spécifique du CPS5 tout en contrôlant les variations temporelles et les facteurs contextuels [16,20]. Les résultats ont été présentés sous forme de tableaux et graphiques.

### Considérations éthiques

L'utilisation des données de routine a été autorisée par les autorités sanitaires locales, et toutes les informations ont été traitées de manière anonyme, sans identification des patients. Aucun consentement individuel n'a été requis, conformément aux réglementations locales pour les études utilisant des données de routine.

## Résultats

### Analyse descriptive

**Figure 2:**
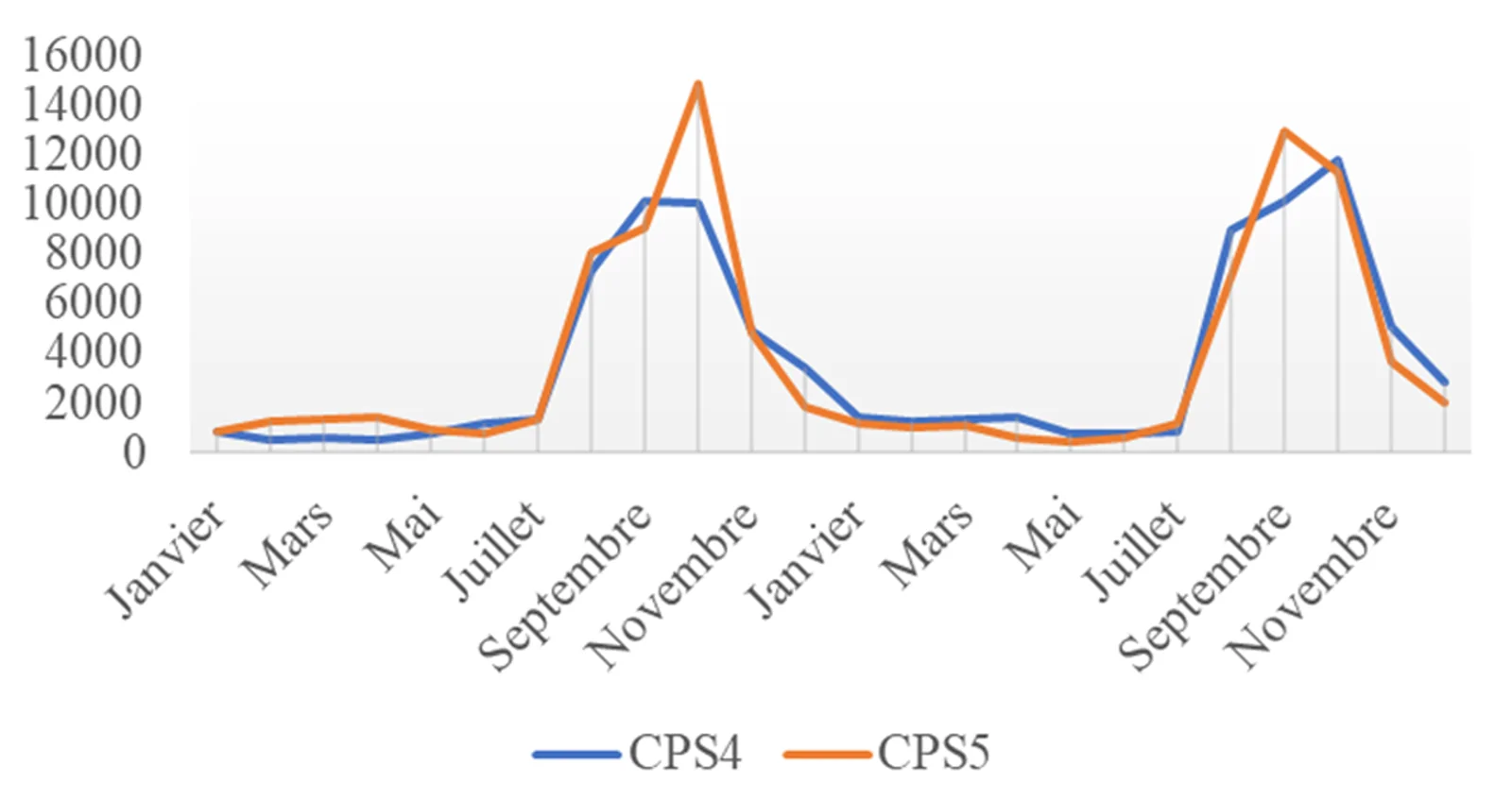
évolution de cas de paludisme avant et après l´intervention au district sanitaire de Takieta

Après analyse des résultats, on observe une couverture élevée avant et après l'intervention, avec une légère progression en post-intervention. En revanche, la couverture dans la zone témoin est plus faible. Concernant les résultats des TDR, on note une diminution de 64% à 54% dans la zone d´intervention, et une augmentation dans la zone témoin, de 78% à 93,44% après l'intervention. Quant à l'utilisation des ACT, elle présente une diminution dans les deux zones (Tableau 1, Tableau 2). Nous observons également une diminution progressive du nombre de cas de paludisme durant le mois de novembre (mois de l´extension), lorsque nous comparons les données avant et après l´intervention dans les deux zones (Figure 2). En calculant le taux de variation de l'incidence avant et après l´extension d´un cinquième passage, on obtient une réduction de 24,5% (Tableau 3). En effet, la simple observation du changement avant et après la mise en œuvre de l'intervention ne suffit pas à mesurer son effet causal. En effet, de nombreux autres facteurs variables dans le temps peuvent l'influencer, d'où l'importance d´utiliser la méthode de la double différence. Le taux de réduction est obtenu par la formule suivante:


$$\frac{\left(\text{TOTAL CAS PALUDISME AVANT}-\text{TOTAL CAS PALUDISME APRES}\right)=100}{\text{TOTAL CAS PALUDISME AVANT}}$$

Calcul de réduction = (16115-12151)/16115 = 24,5%

**Tableau 1:** double de différence

Statut	Période 2020-2021(Pré)	Période 2022-2023 (Prost)	Différence (post-pré)
Districts participants	B	A	B - A
Districts non participants	D	C	D - C
Différence	B - D	A - C	DD= (B - A) - (D - C)

**Tableau 2:** répartition des indicateurs clés en fonction de la période et par zone

Zones	Période	Cible CPS	Enfants ayant reçus tous les passages	TDR+(n)	Total Tests (n)	ACT Administrés (n)	%TDR+ [IC 95%]	%Couverture CPS [IC 95%]
**Intervention**	Avant	69112	68 491	41665	62187	44257	67,00 [66,6;67,4]	99,10[99,06;99,14]
	Après	69112	68751	41237	75980	39831	54,27 [53,9;54,6]	99,47 [99,44;99 ,50]
**Témoin**	Avant	109020	102 482	53 257	67577	72232	78,81 [78,5;79,1]	94,00 [93,93;94,07]
	Après	109020	93523	51198	54919	33474	93,22 [92,9;93,6]	85,78 [85,78;88,88]

**Tableau 3:** cas de paludisme en novembre-décembre dans le district sanitaire de Takieta (2020-2023): analyse pré, per et post-intervention

Période	Année	Cas (novembre)	Cas (décembre)	Total (nov-déc)
Pré intervention	2020	4904	3351	8255
Pré intervention	2021	5038	2822	7860
Intervention	2022	4813	1921	6642
Post-intervention	2023	3588	1829	5509

### Modèle de doubles différences à effet fixe

**Figure 3:**
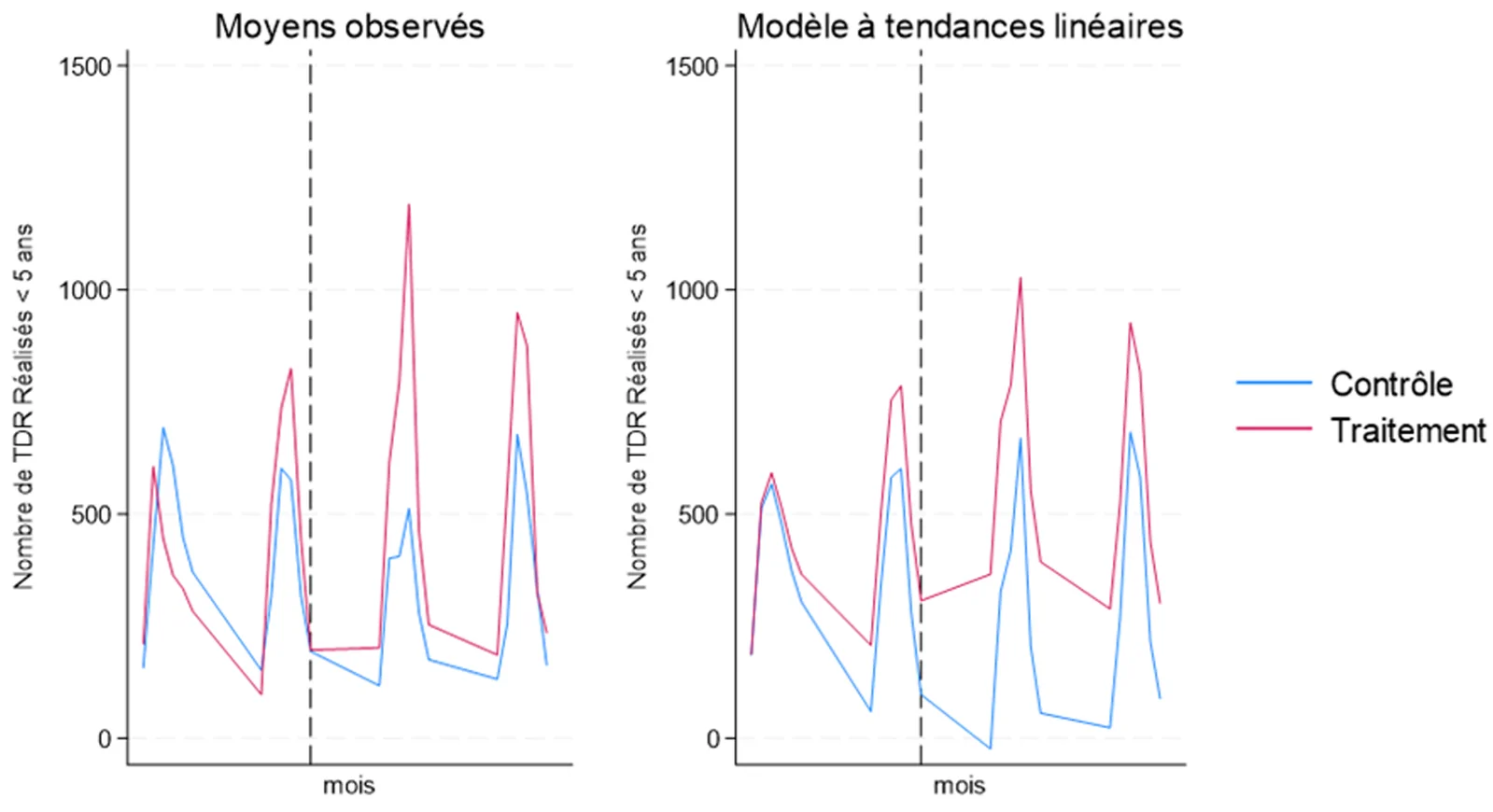
diagnostic graphique des tendances parallèles sur les nombres de TDR réalisés

**Figure 4:**
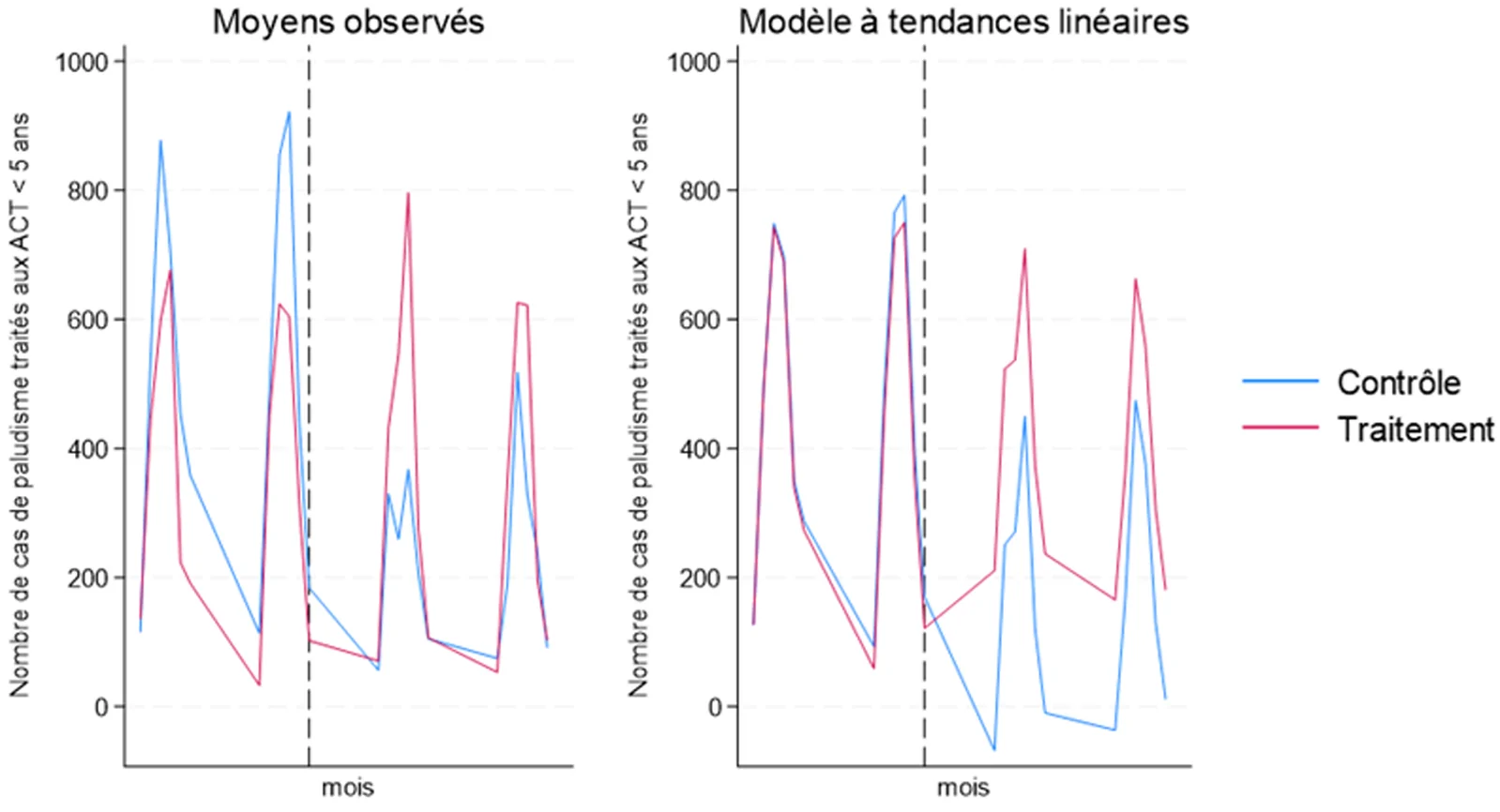
diagnostic graphique des tendances parallèles sur le nombre cas de paludisme traités aux ACT

L'analyse (Tableau 4) montre qu'avant l'extension de la CPS à un cinquième passage mensuel, les deux groupes de districts (traités et contrôles) présentaient respectivement des taux d´incidence de 380,0 et 286,2 pour 1 000, soit une différence significative de 93,8 pour 1 000. En ce qui concerne l'utilisation des services curatifs, on constate plutôt une augmentation. On observe également que les coefficients de double différence pour l'utilisation des TDR et des ACT sont différents de zéro, avec respectivement 158,5 et 153,0 (Tableau 5). L'une des hypothèses de ce modèle est que les trajectoires de l'utilisation des TDR sont parallèles pour les groupes de contrôle et de traitement avant la mise en œuvre de la CPS5 (Figure 3). Le test et l'analyse graphique confirment l'hypothèse de tendances parallèles pour l'utilisation des ACT (Figure 4).

**Tableau 4:** taux d'incidence moyenne et de cas paludisme et d´utilisation de service curative selon le statut CPS du district et la période

Zone	Taux d'incidence			Taux d'utilisation de service curative		
	Après	Avant	Différence	Après	Avant	Différence
Zone participant (CPS5)	383,77	380,09	3,68	46,18	40,59	5,59
Zone non-participant (CPS4	280,16	286,28	-6,12	46	43,4	2,6
Différence	103,61	93,8	**-2,44**	0,18	-2,81	**2,99**

**Tableau 5:** le nombre moyenne d'utilisation des TDR et des ACTs selon le statut CPS du district et la période

Zone	Nombres des TDR utilisés			Utilisation des ACTs		
	Après	Avant	Différence	Après	Avant	Différence
Zone participant (CPS5)	395,72	286,49	109,23	208,53	208,759	-0,22
Zone non-participant (CPS4)	218,8	268,16	-49,36	133,36	286,63	-153,27
Différence	176,92	18,33	**158,59**	75,17	-77,87	**153,05**

## Discussion

Cet article est rédigé dans le but de déterminer l'impact de l'ajout d'un cycle mensuel de la chimioprévention du paludisme sur l'utilisation des tests de diagnostic rapide (TDR), l'utilisation des combinaisons thérapeutiques à base d'artémisinine (ACT) et la réduction de l´incidence du paludisme. Dans un premier temps, nous avons utilisé une évaluation avant-après pour déterminer l´effet de cette intervention sur les indicateurs clés de notre étude. Cela nous a permis d'observer une couverture élevée dans la zone d'intervention, ce qui pourrait indiquer une bonne organisation et une bonne acceptation du programme. Ces résultats sont supérieurs à la recommandation de l´Organisation mondiale de la Santé (OMS), qui est de 80% [6]. Les TDR positifs diminuent significativement dans la zone d'intervention, tandis qu'ils augmentent dans la zone témoin. Cela suggère une réduction probable de l´incidence du paludisme dans la zone d´intervention, bien qu´elle ne soit pas mesurée directement. On note également une diminution progressive des cas de paludisme au cours du mois où l'intervention a eu lieu.

Durant les deux années d´intervention, nos résultats sont inférieurs à ceux trouvés par Diarra [21], qui a rapporté une incidence de cas de paludisme de 84,40% durant la période standard, contre 82,90% durant la période d'intervention. Le nombre de cas de paludisme pendant la période sans intervention était plus élevé que celui observé durant la période d´intervention [21]. En revanche, nous avons observé une réduction significative de l'incidence du paludisme de 24,59%. Cette réduction est corroborée par une baisse du nombre de TDR positifs. Ces résultats sont supérieurs à ceux d´une étude de cohorte menée au Mali en 2020 sur l´effet d'un cinquième passage, qui a montré une diminution de 19% [22]. Cette différence pourrait s´expliquer par la méthodologie utilisée dans un premier temps, qui comportait des biais méthodologiques. Cependant, nous avons eu recours à la méthode de la double différence (DiD), qui nous a permis de mesurer l'effet spécifique de l'intervention [20]. Cette analyse permet de contrôler les biais potentiels liés aux différences préexistantes entre les groupes cas et témoins, ainsi qu'aux tendances temporelles externes. Des différences ont également été observées par rapport à d´autres études menées dans des contextes similaires. Ainsi, après analyse des résultats de cette intervention, on constate une réduction significative de l'incidence de 2,44%, ce qui concorde avec une étude d'essai randomisé ouvert réalisée entre juillet et décembre 2020 au Mali, qui a trouvé une réduction significative des cas de paludisme dans le groupe d´intervention [23]. Ces variations peuvent s´expliquer par plusieurs facteurs, tels que les caractéristiques de la population, la méthode de collecte des données et les conditions d'étude. En ce qui concerne l'utilisation des TDR, les deux groupes de districts (traités et contrôlés) présentaient, avant l'extension, des moyennes respectives de 286,49 et 268,16, soit une différence significative de 18,33. Le coefficient de la double différence (Diff-in-Diff) est significatif et différent de zéro, ce qui signifie que l´ajout d´un passage mensuel a modifié l´utilisation des TDR. En particulier, l´utilisation des TDR a augmenté en moyenne de 158,59 par rapport à ce qu'elle aurait été sans l'ajout d'un cinquième passage mensuel.

Nous remarquons ainsi une réduction de l´incidence au fil des années de mise en œuvre de la CPS, mais l´utilisation des ACT et des TDR ne cesse d´augmenter. Cela peut s'expliquer par plusieurs raisons, notamment le fait que, dans les zones d'endémie, les TDR permettent d'éviter l'utilisation systématique de traitements présomptifs, ce qui contribue à la sélection de souches résistantes [24], ou encore par une mauvaise utilisation des TDR. Il faut également noter que l'utilisation des ACT, avant l'extension, dans les deux groupes de districts (traités et contrôlés), présentait respectivement une moyenne de 208,75 et 286,63, soit une différence de 77,87. Le coefficient Diff-in-Diff est significatif et différent de zéro, ce qui signifie que l'ajout d'un passage mensuel a modifié l'utilisation des ACT. L´utilisation des ACT a ainsi augmenté en moyenne de 153,05 de plus que ce qu´elle aurait été sans ce cinquième passage mensuel.

Notre étude a également révélé que l'intervention n´a pas eu d'impact sur le taux de consultation. Cela peut probablement expliquer l´augmentation de l'utilisation des TDR, car tout enfant de 0 à 5 ans suspecté de paludisme reçoit un test de dépistage rapide gratuit. En revanche, nous avons trouvé que l´intervention a un lien statistiquement significatif avec la positivité des TDR, et qu'en cas de test positif, un traitement antipaludique par voie orale à base d'ACT doit être administré [25]. Il est fort probable que l´absence de réduction de l'utilisation des ACT soit liée au non-respect des directives de prise en charge du paludisme [25]. Ces mêmes observations ont été faites dans une étude d'estimation précise de la charge du paludisme au Niger, dont les résultats ont mis en évidence des insuffisances dans la description de la situation du paludisme et du risque de maladie, en raison d'un surdiagnostic chez les patients présentant une fièvre simple. La confirmation des cas nécessitera donc un changement de comportement, notamment à travers la formation du personnel soignant, l´instauration d'un contrôle de qualité, une supervision accrue des centres de santé intégrés, la mise en œuvre de bonnes pratiques cliniques, et une meilleure utilisation des outils de diagnostic disponibles [26].

Bien que nos résultats présentent une meilleure efficacité pour la stratégie, quelques limites méthodologiques peuvent être considérées, surtout l'utilisation de TDR positif bien qu´il soit un indicateur indirect de la mesure de transmission du paludisme. L'étude avait inclus certains facteurs tels que la durée d´observation qui est courte (2 ans) et ne permet pas d´évaluer l´impact à long terme. L´absence de données sur les résistances parasitaires locales. D'autres biais liés aux données de routine comme ces données basées sur la surveillance passive. De plus, la modélisation de type DD est basée sur une agrégation de données à l´échelle de districts de santé, ce qui constitue également une limite, car des spécificités propres aux centres de santé peuvent se trouver diluées dans le groupe [16].

## Conclusion

L'ajout d'un cinquième cycle mensuel de CPS a permis de réduire l'incidence du paludisme et le nombre de tests rapides positifs dans les districts étudiés, ainsi que d'améliorer légèrement la fréquentation des consultations curatives. En revanche, aucune réduction significative de l´utilisation globale des TDR ni des ACT n´a été observée. Ces résultats montrent que l'impact de la CPS5 s´est manifesté sur la diminution de l'incidence du paludisme et des TDR positifs, mais pas sur la consommation des services diagnostiques et thérapeutiques.
